# Remote impacts of 2009 and 2015 El Niño on oceanic and biological processes in a marginal sea of the Northwestern Pacific

**DOI:** 10.1038/s41598-021-04310-8

**Published:** 2022-01-14

**Authors:** Yoonho Jung, Jae-Hun Park, Naoki Hirose, Sang-Wook Yeh, Kuk Jin Kim, Ho Kyung Ha

**Affiliations:** 1 Department of Ocean Sciences, Inha University, Incheon, 22212 South Korea; 2 Research Institute of Applied Mechanics, Kyushu University, Fukuoka, 816-8580 Japan; 3 Department of Environmental Marine Science, Hanyang University, Ansan, 15588 South Korea; 4 Underwater Survey Technology 21, Incheon, 21999 South Korea

**Keywords:** Physical oceanography, Ocean sciences

## Abstract

The significance of long-term teleconnections derived from the anomalous climatic conditions of El Niño has been a highly debated topic, where the remote response of coastal hydrodynamics and marine ecosystems to El Niño conditions is not completely understood. The 14-year long data from a ship-borne acoustic Doppler current profiler was used to examine the El Niño’s impact, in particular, 2009 and 2015 El Niño events, on oceanic and biological processes in coastal regions across the Korea/Tsushima Strait. Here, it was revealed that the summer volume transport could be decreased by 8.7% (from 2.46 ± 0.39 to 2.24 ± 0.26 Sv) due to the anomalous northerly winds in the developing year of El Niño. Furthermore, the fall mean volume backscattering strength could be decreased by 1.8% (from − 97.09 ± 2.14 to − 98.84 ± 2.10 dB) due to the decreased surface solar radiation after the El Niño events. Overall, 2009 and 2015 El Niño events remotely affected volume transport and zooplankton abundance across the Korea/Tsushima Strait through climatic teleconnections.

## Introduction

Climate change has major effects on atmospheric and oceanic conditions, leading to changes in precipitation, wind, and ocean currents^1,2^. In particular, the El Niño-Southern Oscillation (ENSO) is the most dominant climate signal that affects the global climate with anomalies in the sea surface temperature (SST) and wind system^3^. Previous observations indicated that the offshore SST and climate signals across East Asia are closely related to these El Niño patterns despite the long distance from the Central Pacific^4,5^. Hence, the climate is generally warmer and wetter in East Asia during an El Niño winter and subsequent spring. Because climate variability in the open ocean affects regional areas, their associated teleconnection has been highlighted to predict the interannual variability of regional marine ecosystems. Ref.^6^ showed that the timing of the vertical migration and the abundance of zooplankton under seasonally varying sea ice correlated with the ENSO in the Amundsen Sea, Antarctica. A close relationship was observed between the El Niño seasons and East Asian regions (Korea, Japan, China and Taiwan) biomass, where differences in the SST anomaly (SSTA) and precipitation patterns lead to changes in the biomass location following the latitudinal migration of the salinity front^7^.

**Figure 1:**
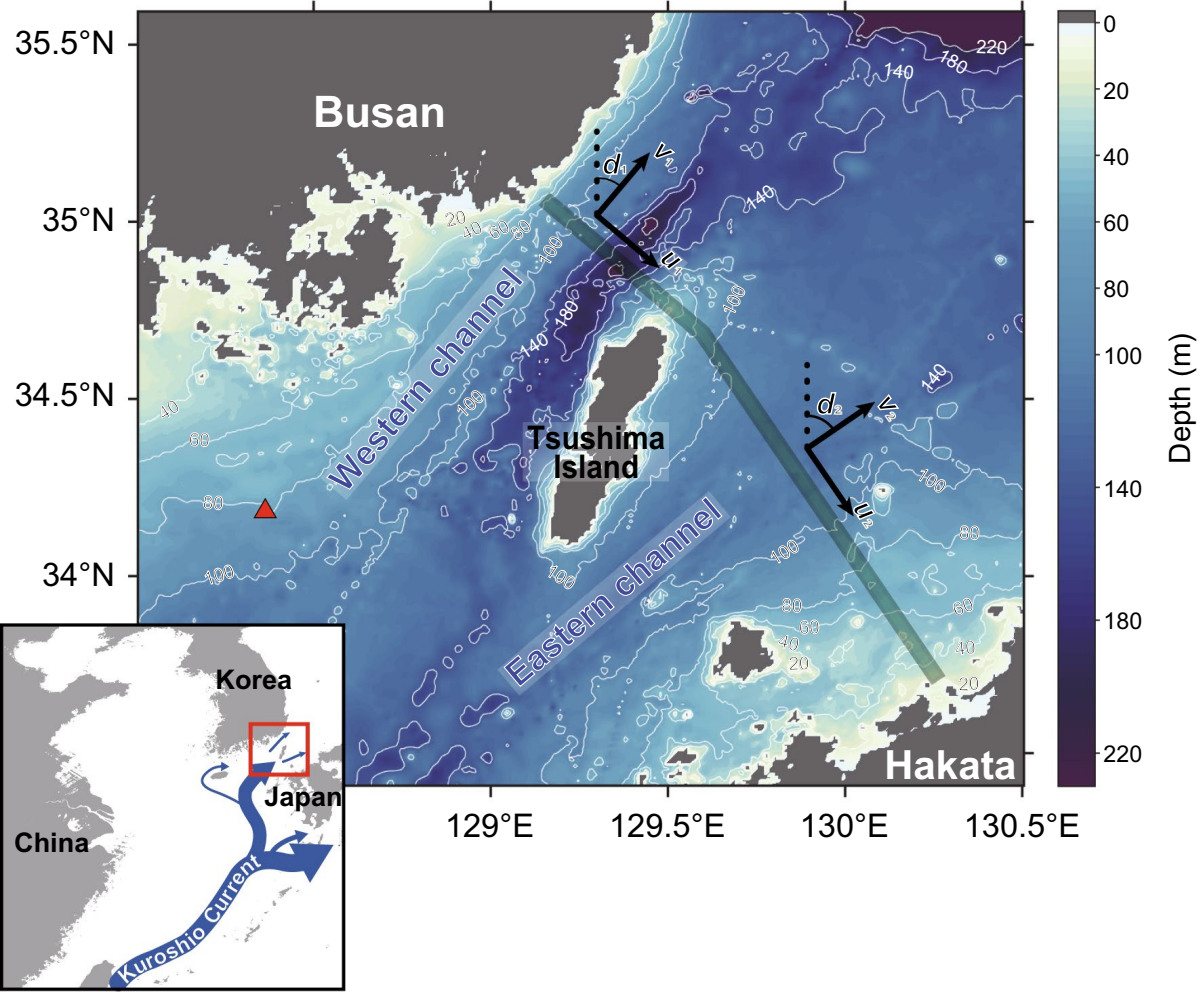
Study area map of the Korea/Tsushima Strait. The green shaded area includes 95% of the ferry boat (*New Camellia*) trajectories. *u* and *v* represent the along- and across-track velocities where subscripts 1 and 2 indicate the western and eastern channels, respectively. *d*_1_ and *d*_2_ are the angles relative to North (40° and 56°). The red triangle represents the location of the bottom-moored ADCP (Supplementary Fig. 5). The contour intervals are 20 m (0**–**100 m) and 40 m (> 100 m). The figure was created using MATLAB (ver. 9.9.0.1592791 (R2020b) Update 5). The bathymetry is based on the SRTM30_PLUS dataset (https://topex.ucsd.edu/).

Although the relationship between the local oceanic processes and climatic factors has been highlighted, the response of coastal hydrodynamics and marine ecosystems to El Niño is not completely understood^8–10^. For example, the coastal hydrodynamic models included complex interactions between Pacific basin-scale wind forcing and local wind stress in order to predict the seasonal cycle of volume transport, but did not include interannual climate variabilities^11^. Ref.^10^ suggested that the typhoon-dependent nutrient supply largely regulates the abundance and composition of the phytoplankton assemblages. These studies were generally based on short-term (< 2 years) observations rather than long-term monitoring efforts^10^. Therefore, volume transport and zooplankton abundance potentially depend on the poorly understood interplay between oceanic processes and climate systems, and require an in-depth understanding of the impact of El Niño on the local weather and marine ecosystems. This study examined the effects of two El Niño events, which occurred in 2009 and 2015, on the seasonal and interannual variability in volume transport and mean volume backscattering strength (MVBS) from a ship-borne acoustic Doppler current profiler (ADCP) across the Korea/Tsushima Strait (KTS; Fig. 1) as a proxy for coastal hydrodynamics and ecosystems. Because modelling data is generally used to show the overall tendency or mean volume transport so that site-specific long-term measurements are necessary in order to determine the effects of El Niño on the seasonal variations of volume transport and MVBS across the KTS. We have therefore estimated volume transport and MVBS between 2004 and 2017 based on daily observational data. Seasonal comparisons with NINO indices and climate signals were then applied to determine the remote impacts of El Niño on the temporal variability in the ADCP data.

## Results and discussion

### Impacts of El Niño on volume transport and zooplankton abundance

**Figure 2:**
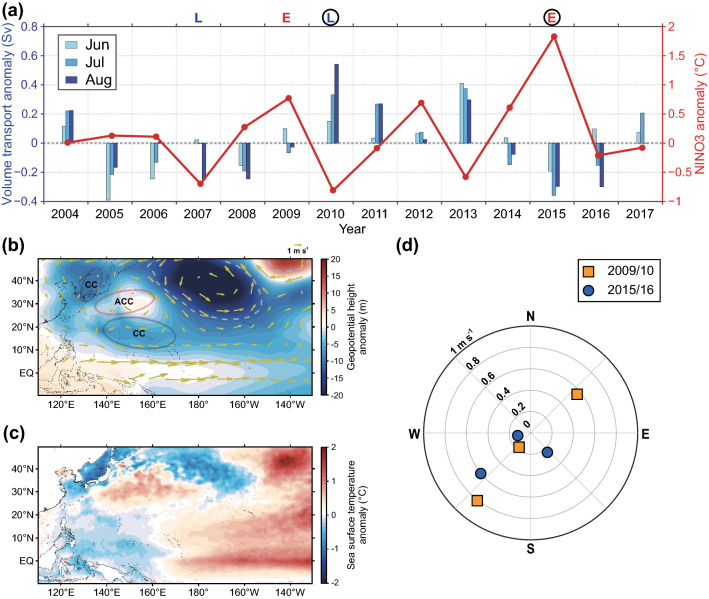
| Remote impact of NINO3 on volume transport through external climatic factors in summer (JJA). (**a**) Volume transport anomaly (blue bars) and averaged NINO3 anomaly (red line) for summer. E and L on top of (**a**) indicate the developing years of El Niño and La Niña events, respectively. Surrounding black circles mean that summer NINO3 and PDO are in phase. (**b**) Composite maps of the averaged anomalies of wind velocities and geopotential height at 850 hPa during the developing summer of El Niño years (2009/10 and 2015/16). CC indicates a cyclonic circulation, and ACC indicates an anti-cyclonic circulation. Gray dashed lines denote statistical significance at the 90% confidence level. (**c**) Composite maps of the averaged anomalies of sea surface temperature during the developing summer of El Niño years (2009/10 and 2015/16). (**d**) Wind velocity anomalies over the Korea/Tsushima Strait during the developing summer of El Niño years. The figure was created using MATLAB (ver. 9.9.0.1592791 (R2020b) Update 5). The coastline is based on the ETOPO1 dataset (https://www.ngdc.noaa.gov/).

The 14-year-long ADCP data across the KTS and climate signals were compared with the NINO indices to examine the possible teleconnection between them (Supplementary Fig. 1). During this period, two El Niño events (2009 and 2015) and two La Niña events (2007 and 2010) occurred. The ADCP and climate data were mostly correlated with NINO3 with a 0 to 9-month lag (Supplementary Table 1). This result indicates that the SSTA in the Eastern Pacific region might be associated with the climate system over the KTS with a lagged time during the study period. Summer volume transport (1.71–3.21 Sv at 95% confidence level) tended to decrease in the developing years of 2009 and 2015 El Niño events (Fig. 2a). Overall, mean volume transport across the KTS tends to be influenced by Pacific basin-scale wind forcing through the pressure difference between the KTS and Tsugaru Straits^12^ as well as the meridional pressure gradient between the subtropical and subpolar gyres in the interior North Pacific Ocean^13^. However, local or seasonal variations in volume transport are rather impacted by local wind forcing over the KTS^11^. Ref.^11^ showed that the ratio between these two contributions is about two to one. Local wind forcing is known for hampering volume transport in the anomalous northerly wind conditions. In northwesterly wind condition around the KTS (Fig. 2b), Ekman transport induces upstream currents. Ref.^14^ showed that the anomalous northeasterly winds decrease volume transport through a combination of across-strait geostrophic and along-strait ageostrophic balances.

Figure 2b shows the composited wind velocity anomalies and geopotential height at 850 hPa during the developing summer of two El Niño years. It is found that the development of a low-pressure system in the western North Pacific (20–45° N, 110–140° E) including the KTS during the developing year of the two El Niño events produced anomalous northerly winds. During this time, the anomalous warm SST in the central-to-eastern tropical Pacific (5° N–5° S, 170° E–130° W) (Fig. 2c) induces a Gill-type response of cyclonic atmospheric circulation in the western-to-central tropical Pacific (10–25° N, 130–170° E)^15^, and concurrently, the anti-cyclonic circulation can be induced in the western subtropical Pacific (20–30° N, 130–160° E) as a secondary circulation^5^. This leads to an anomalous cyclonic circulation in the western North Pacific via atmospheric teleconnections with northerly winds (Fig. 2b). This implies that the climatological southerly winds decreased over the KTS during boreal summer (Fig. 2d), resulting in the weakening of volume transport (Fig. 2a). This result agrees well with previous studies^11,16^ that examined the northerly wind conditions under which volume transport is reduced. Here, the wind stress varies with the East Asian monsoon; the northerly winds in winter generally reduce volume transport, whereas the southerly winds in summer enhance volume transport^11^.

**Figure 3:**
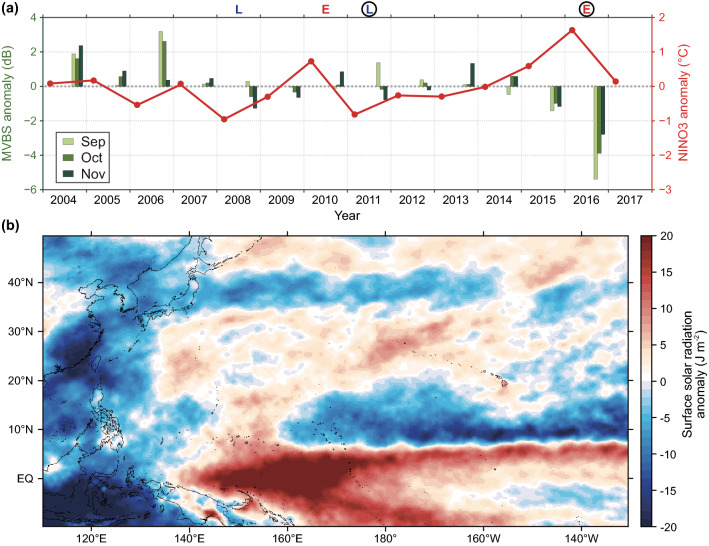
| Remote impact of winter (DJF) and spring (MAM) NINO3 on the fall (SON) mean volume backscattering strength (MVBS) through external climatic factors. (**a**) Fall MVBS anomaly (green bars) and averaged NINO3 anomaly (red line) for winter and spring. E and L on top of (**a**) indicate the decaying years of El Niño and La Niña events, respectively. Surrounding black circles mean that NINO3 and PDO are in phase and maintain their signs from winter to fall. (**b**) Composite maps of the averaged anomalies of surface solar radiation during the decaying fall of El Niño years (2009/10 and 2015/16). The figure was created using MATLAB (ver. 9.9.0.1592791 (R2020b) Update 5). The coastline is based on the ETOPO1 dataset (https://www.ngdc.noaa.gov/).

Ref.^17^ reported that the zooplankton abundance is dependent on light availability because the chlorophyll-*a* concentration is low in limited light regions. Similarly, the fall MVBS (**− **101.28 to  − 92.90 dB at 95% confidence level) decreased by a reduction in surface solar radiation (SSR) (Fig. 3). Low cloud cover and precipitation increased, and the SSR and fall MVBS decreased to below average after the El Niño (Supplementary Table 1; Supplementary Figs. 2 and 3). These analyses suggest that the increased low cloud cover in fall following El Niño reflected solar radiation and limited the surface light. Comparisons between the winter NINO3 and fall MVBS showed that a decrease in fall SSR and an increase in fall surface thermal radiation after an El Niño event were responsible for reducing the fall MVBS (Supplementary Table 1). Similarly, previous work^18^ found that the SSR and thermal radiation have generally opposing trends, and a decreased SSR indicates an increase in cloud coverage. The fall MVBS then decreased concomitantly with a reduction in zooplankton abundance.

We further examined the relationship between MVBS and zooplankton abundance over timescales long enough to identify the seasonal patterns and the El Niño-driven interannual variability of both parameters (Supplementary Figs. 4 and 5). MVBS is commonly used as a proxy for local zooplankton biomass, but a single frequency ADCP cannot accurately identify the species responsible for the acoustic backscatterer^6^ because the detectable particle size exceeds the size of plankton^19^. Despite this potential complication, the spatial and temporal variability in zooplankton abundance can be predicted from the magnitude of phytoplankton blooms^20^. The positive relationship between MVBS and the chlorophyll-*a* concentration indicates the capability of MVBS to detect zooplankton (Supplementary Fig. 4). Furthermore, the diel vertical migration pattern visible in the bottom-moored ADCP data is strong evidence of the existence of zooplankton across the KTS and the capability of 300-kHz ADCP to detect the abundance of zooplankton (Supplementary Fig. 5). Ref.^21^ used the Bray–Curtis cluster analysis to compare the zooplankton abundance between two stations near the western channel. They reported that the species composition and abundance were similar in both sampling locations. The bottom-moored ADCP data (for location, see red triangle in Fig. 1) shows the typical pattern of diel vertical migration, where a low MVBS appears near the surface during the day and a high MVBS occurs near the surface during the night (Supplementary Fig. 5). A similar pattern can be recognized from the *New Camellia* MVBS, despite missing data due to the regular fixed operation schedule of the ferry.

**Figure 4:**
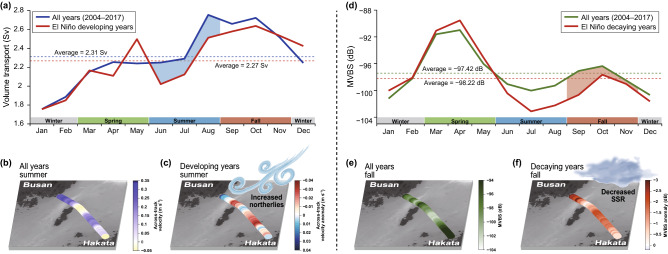
Climatic impact of El Niño on volume transport and the mean volume backscattering strength (MVBS) across the Korea/Tsushima Strait. (**a**) Monthly average of volume transport during all years and El Niño developing years (2009 and 2015). The shaded area indicates the difference in summer (JJA) volume transport between the two time windows. (**b**) Averaged summer across-track velocities during all years. (**c**) Averaged summer across-track velocity anomalies during El Niño developing years. (**d**) Monthly average MVBS during all years and El Niño decaying years (2010 and 2016). The shaded area indicates the difference in fall (SON) MVBS between the two time windows. (**e**) Averaged fall MVBS during all years and (**f**) Averaged fall MVBS anomalies during El Niño decaying years. The figure was created using MATLAB (ver. 9.9.0.1592791 (R2020b) Update 5). The bathymetry is based on the SRTM30_PLUS dataset (https://topex.ucsd.edu/).

Although seasonal variations in volume transport and MVBS reveal a distinct pattern over the entire observation period (Fig. 4), the effects of El Niño on the two parameters are only visible in the summer and fall, respectively (Supplementary Table 1). On the other hand, there was a significant relationship between winter and spring volume transport and the SST, suggesting that the heat transport across the KTS depends largely on the local volume transport during the cool period (Supplementary Table 2). Additionally, a negative correlation between the spring MVBS and SST suggests that the spring chlorophyll-*a* concentration decreased (increased) with increasing (decreasing) SST (Supplementary Table 2). This contradicts previous work, where the light availability and the resulting increases in temperature during the spring months are responsible for a phytoplankton bloom during the same period^22^. This suggests that local spring biomass distribution may not be controlled by atmospheric processes alone. Instead, oceanic processes such as nutrient availability, are more likely to influence the zooplankton behavior^23^. Furthermore, SSTA tends to naturally phase out as part of the seasonal cycle, which weakens the influence of warming in the tropical region. The mean seasonal cycle without changes in the SSTA may be responsible for the absence of a significant relationship between the NINO indices and spring MVBS^10,24^.

### Limitations and further discussion

The temporal extent of the study period explains the potential limitations of the statistical significance of these results. Because El Niño commonly occurs on longer timescales (Eastern Pacific El Niño (EP-El Niño): 3–7 years; Central Pacific El Niño (CP-El Niño): 2–3 years, 10–15 years^3^), El Niño research focuses mostly on longer time series (> 30 years). To alleviate this concern, the climatic impacts on the ocean are compared in terms of the superimposed impacts of two El Niño events, instead of dividing them into the effects of separate EP/CP-El Niño. La Niña events are also a potential factor for determining hydrodynamic and biological processes across the KTS. Because the impacts of EP- and CP-El Niño on volume transport tend to be stronger in amplitude, and exhibit more pronounced spatial diversities^3^, La Niña events were excluded from this study. The arguments are based mainly on a series of statistical analyses.

The comparisons underscore the relative importance of the influence of EP-El Niño (i.e., 2015 El Niño) on the East Asian oceanic variances compared to CP-El Niño (i.e., 2009 El Niño). Ref.^3^ showed that EP-El Niño is generally associated with basin-scale equatorial winds and large eastward shifts of the tropical Pacific convection zone, as well as the strong discharge of the heat content from the equatorial band. In contrast, CP-El Niño is associated more commonly with local wind feedback, weak shifts of the convection zone, earlier termination, and only limited discharge of the ocean heat content. This supports the present findings that volume transport and MVBS were lowest when the NINO3 was the highest (2015 El Niño) during the entire analyzed period. On the other hand, Ref.^25,26^ further argued that the response of ENSO over East Asia is strong when the ENSO-Pacific Decadal Oscillation (PDO) relationship is “in phase”, i.e., either El Niño and a positive PDO phase or La Niña and a negative PDO phase, compared to that when the ENSO-PDO is “out of phase”. This is because the atmospheric response over the western tropical Pacific is enhanced when the ENSO and PDO are in phase in which the SSTA has the same sign in the central-to-eastern tropical Pacific. In fact, the impact of El Niño on volume transport and MVBS could be enhanced, when NINO3 and PDO are in phase (Figs. 2a, 3a, and Supplementary Fig. 6). Summer volume transport significantly decreased when both indices were positive (2015 El Niño), whereas such phenomenon is weak when El Niño and PDO are out of phase (2009 El Niño). Similar results were also obtained when two La Niña events (2007 and 2010) were compared with the PDO index (Figs. 2a and 3a). However, this finding is limited to a single case of ENSO event, so that it is necessary to further examine whether a constructive and destructive influence depending on the ENSO-PDO relationship on volume transport is statistically significant.

## Conclusions

The impacts of low-latitude climate systems on the interannual variability including ENSO events on local volume transport and zooplankton is not completely understood. This study found that El Niño caused an 8.7% (from 2.46 ± 0.39 to 2.24 ± 0.26 Sv) decrease in volume transport and a 1.8% (from − 97.09 ± 2.14 to − 98.84 ± 2.10 dB) decrease in MVBS due to anomalous northerly winds and anomalously increased cloud coverage, respectively, indicating that there may exist a linkage between El Niño and oceanic responses through atmospheric teleconnection. Therefore, the hydrodynamics and marine ecosystems across the KTS could be sensitive to low-latitude climate signals including ENSO events. This suggests that the changes in physical background in the tropical Pacific will have major impacts on the weather and marine ecosystems through variations in volume transport and zooplankton abundance within the East Asian regions. In particular, oceanic responses were significant when NINO3 and PDO were in phase, so that SSTA has the same sign in the central-to-eastern tropical Pacific. Our results further underscore the relative importance of the influence of EP-El Niño on the East Asian oceanic variances compared to CP-El Niño. Overall, the changes in volume transport and MVBS are a good proxy for the teleconnection between El Niño and the mid-latitude oceanic processes.

## Methods

### Kuroshio current and Korea/Tsushima Strait

The Tsushima Warm Current is a branch of the Kuroshio Current that originates east of the Philippine coast by the bifurcation of the North Equatorial Current^27^. The Kuroshio Current flows northward along the continental shelf break, where mixing processes between the East China Sea and the Kuroshio Current occur^28^. The Kuroshio Current consists mainly of three water masses (Kuroshio Surface Water, Kuroshio Tropical Water, and Kuroshio Intermediate Water) with different origins and nutrient properties; hence, the characteristics of each part of the Kuroshio Current depend on the mixing rate^28^. The Kuroshio Current detaches from the continental shelf break at 30° N and 128–129° E, where it meets cold, fresh continental shelf water and forms a density front and frontal meanders. Subsequent mixing with continental shelf water then forms the origin of the Tsushima Warm Current^27^.

The KTS is a complex channel system with a width and length of 180 km and 330 km, respectively (Fig. 1). This is an important pathway of the Tsushima Warm Current, which transports heat, nutrients, and organic matter from the East China Sea to the East/Japan Sea^8–10^. The Tsushima Warm Current is divided by the Tsushima Islands into the western and eastern channels. The western channel has a narrow and hollow shape with a width of 40 km and a depth of 150–200 m, while the eastern channel is relatively wide and shallow, reaching widths and depths of 140 km and 110 m, respectively^29,30^. Volume transport through the western channel flows northeast along the east coast of the Korean Peninsula, while a deep southwestern countercurrent introduces cold water to the bottom layers^27^.

### Volume transport and mean volume backscattering strength

Long-term current data has been collected with a 300-kHz ADCP mounted on the ferry boat *New Camellia*, operating daily between Busan, Korea, and Hakata, Japan from July 2004 to June 2017 (Fig. 1). The ADCP data was measured at ~ 30 s and 8 m intervals from an ~ 18 m depth below the sea surface to the bottom. A slant angle of 20° produces a strong echo contaminating the signal across the seafloor layer; accordingly, 6% of the near-bottom data were excluded from further analyses^9^. The ADCP data were then averaged into 195 sections with a 1 km interval along the main trajectory of the ferry boat. This approach included 95% of all trajectories between 2004 and 2017 (Fig. 1), and removed outliers for further analyses^9,31^. Because the time intervals between the individual measurements were not consistent in each section, the datasets were merged and interpolated to obtain a continuous daily time series.

Because the KTS is a narrow, shallow strait, tidal currents can reach up to 1 m s^−1^ in the surface layer and exceed 0.5 m s^−1^ at intermediate depths^29^. Therefore, the velocity data includes strong tidal currents, so it is necessary to eliminate the tidal components from the total velocity^32^. The observed current velocities are the sum of the harmonics as follows:

$$v = v_{0} + \mathop \sum \limits_{i = 1}^{16} \left( {a_{i} \sin w_{i} t + b_{i} \cos w_{i} t} \right)$$

where $w_{i}$ ($i$ = 1, 2,…, and 16) is the angular frequency of ten major tidal constituents (*Q*_1_, *O*_1_, *P*_1_, *K*_1_, *N*_2_, *M*_2_, *S*_2_, *K*_2_, *MSf*, and *Mf*) and four additional tidal constituents ($\mu$_2_, *NO*_1_, $\Phi$_1_, and *J*_1_) as well as annual ($i$ = 15) and semiannual ($i$ = 16) variations of the residual currents^9,32^. The variable $t$ is the data measuring time and $v_{0}$ is the residual current without annual and semiannual variations. Thirty-three unknown values ($v_{0}$, $a_{i}$, $b_{i}$ ($i$ = 1, 2,…, and 16)) were estimated using the robust least squares method^29,32^. Volume transport calculations used across-track velocities normal to the *New Camellia* trajectory (Fig. 1).

The acoustic backscatter strength was extracted for each depth bin from the ADCP to calculate MVBS by applying a simplified sonar Eq.^33^. The chlorophyll-*a* concentration (mg l^−1^) collected by *New Camellia* in 2016 was compared with MVBS during the same period using linear regression. Furthermore, *New Camellia* MVBS was compared with the MVBS calculated from the bottom-moored ADCP data, which was collected between August 8th and 17th, 2014 by an up-looking 300-kHz ADCP mounted on a trawl-resistant bottom mount (Fig. 1). The ADCP bin size was 3 m, and the data was measured from ~ 8 m above the bottom to the surface (~ 80 m). Both *New Camellia* and bottom-moored ADCP data were then averaged every hour and compared to detect the diel vertical migration patterns in the MVBS profiles. The *New Camellia* ADCP data were extracted for the western channel at a depth of 70–90 m, covering the same data period as the bottom-moored ADCP data (August 8–17th, 2014).

### El Niño occurrence

The ADCP data were compared with NINO and PDO indices from 2004 to 2017 to account for the spatial variability in atmospheric teleconnection throughout the tropical Pacific region (https://www.esrl.noaa.gov/). NINO3 indicates the mean-removed and 3-month moving averaged SSTA over the 5° N–5° S and 150–90° W region, which indicates the EP-El Niño events. El Niño (La Niña) events are identified by NINO3 exceeding (being less than) 0.5 °C (− 0.5 °C) for more than 8 months^34^. In order to compare NINO indices with climate and ADCP data, composite analyses and 3-month moving average were then applied.

### Climate data and seasonal analysis

The climate data was provided as an ERA5 dataset by the European Centre for Medium-Range Weather Forecasts (ECMWF). The dataset included the following: the SST, sea level pressure, 10 m-above zonal and horizontal wind speeds, precipitation, high cloud cover, medium cloud cover, low cloud cover, total cloud cover, surface thermal radiation, and SSR. Each dataset was normalized to the standard deviation because the MVBS, volume transport, and climate data largely depend on the time of the year. Composite analysis was then applied to describe the yearly anomalies without the average of each calendar month. The 3-month moving averaged composite values were then averaged and divided into four groups based on the season. In this paper, winter is defined as December of the previous year, January and February; spring is defined as March, April, and May; summer is defined as June, July, and August; fall is defined as September, October, and November. Note that all the indices were compared based on their anomalous state. The climate data was extracted between 33.5–35.5° N and 127.75–130.25° E to compare to the NINO indices. Supplementary Table 3 shows the whole sets of correlations between NINO indices and other factors (ADCP and climate data). Because El Nino events consist of a developing and decaying year, the correlations between each factor and season were calculated for each year and the following year (e.g., correlation between winter NINO indices (year 0) with winter, spring, summer, and fall ADCP data (year 0, + 1)), resulting in a total of 1,344 correlations. Additionally, ADCP and climate data were compared (Supplementary Table 2) within the same season (e.g., correlation between winter volume transport and winter precipitation). The following correlations were excluded: (1) only NINO indices and ADCP data were significantly correlated, but no correlation with climate data; (2) only NINO indices and climate data were significantly correlated, but no correlation with ADCP data; (3) NINO indices are significantly correlated with ADCP and climate data, but the same ADCP and climate data are not significantly correlated; (4) either climate or ADCP data occur before NINO indices (e.g., spring ADCP data (year 0) and summer NINO index (year 0) and the time difference between ADCP or climate data and NINO indices exceeds one year. This means only correlations that were significant on a 0.05 level were further considered. After this procedure, only Supplementary Table 1 remains (i.e., lags of 0, 6 and 9 months).

## Supplementary Information

Supplementary Information.
