# Supplementary material for: Remote impacts of 2009 and 2015 El Niño on oceanic and biological processes in a marginal sea of the Northwestern Pacific
Source: Sci Rep. 2022 Jan 14;12:741. doi: 10.1038/s41598-021-04310-8 (PMC8760326; doi:10.1038/s41598-021-04310-8)
Supplement: Supplementary file 1 — Supplementary Information. [file 41598_2021_4310_MOESM1_ESM.docx]

**Supplementary information**

Supplementary Table 1 | Correlations between NINO3 and other factors (volume transport, mean volume backscattering strength (MVBS) and climate data). The correlations (*r*) were calculated for all datasets, but only seasons with significant (*p*<0.05) relationships between volume transport (or MVBS) and climate data within the same season (Supplementary Table 2) are displayed (C3 in Supplementary Table 3). The climate data include 10 m-above southerly winds, precipitation (PREC), low cloud cover (LCC), surface thermal radiation (STR), and surface solar radiation (SSR).


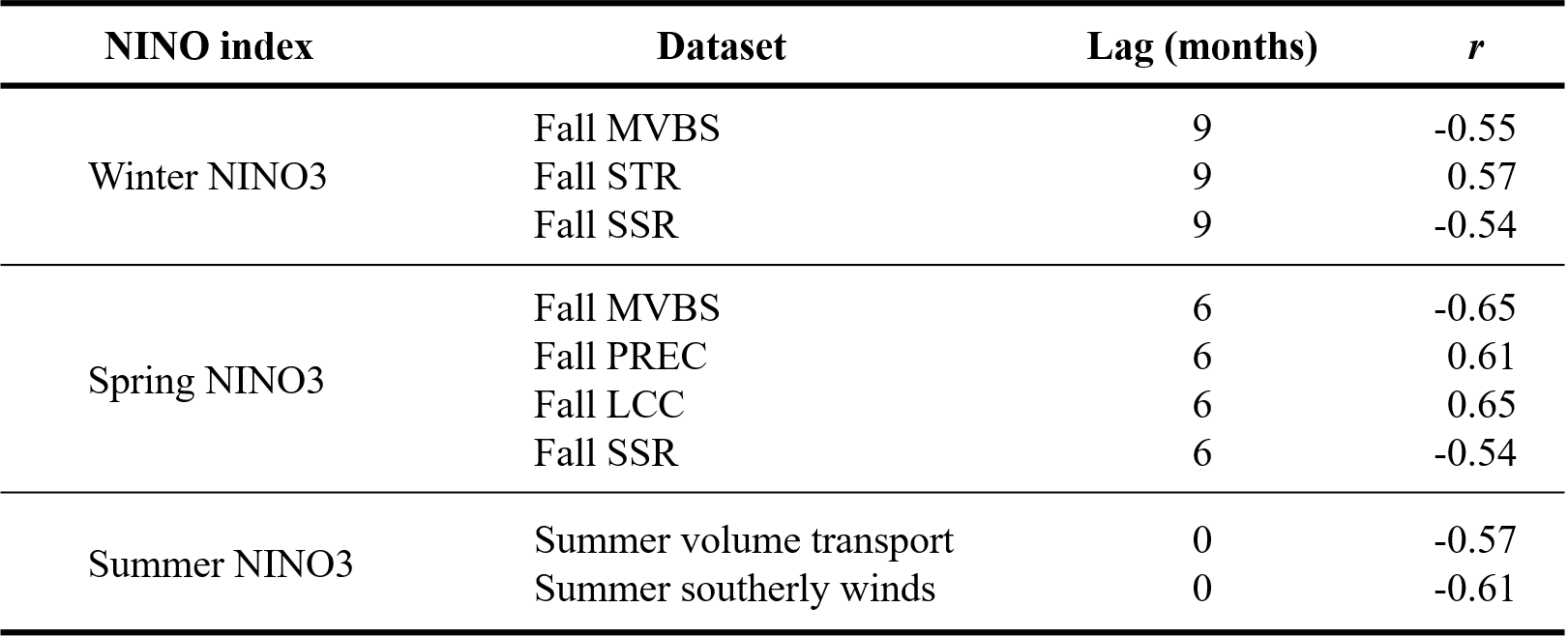


Supplementary Table 2 | Correlations between the acoustic Doppler current profiler (ADCP) data and climate data during the same season. Significant correlations (*r*) between the ADCP data and climate data during the same season are shown (*p*<0.05). ADCP data include mean volume backscattering strength (MVBS) and volume transport, and climate data include the sea surface temperature (SST), 10 m-above southerly winds, precipitation (PREC), high cloud cover (HCC), middle cloud cover (MCC), low cloud cover (LCC), total cloud cover (TCC), surface thermal radiation (STR), and surface solar radiation (SSR).


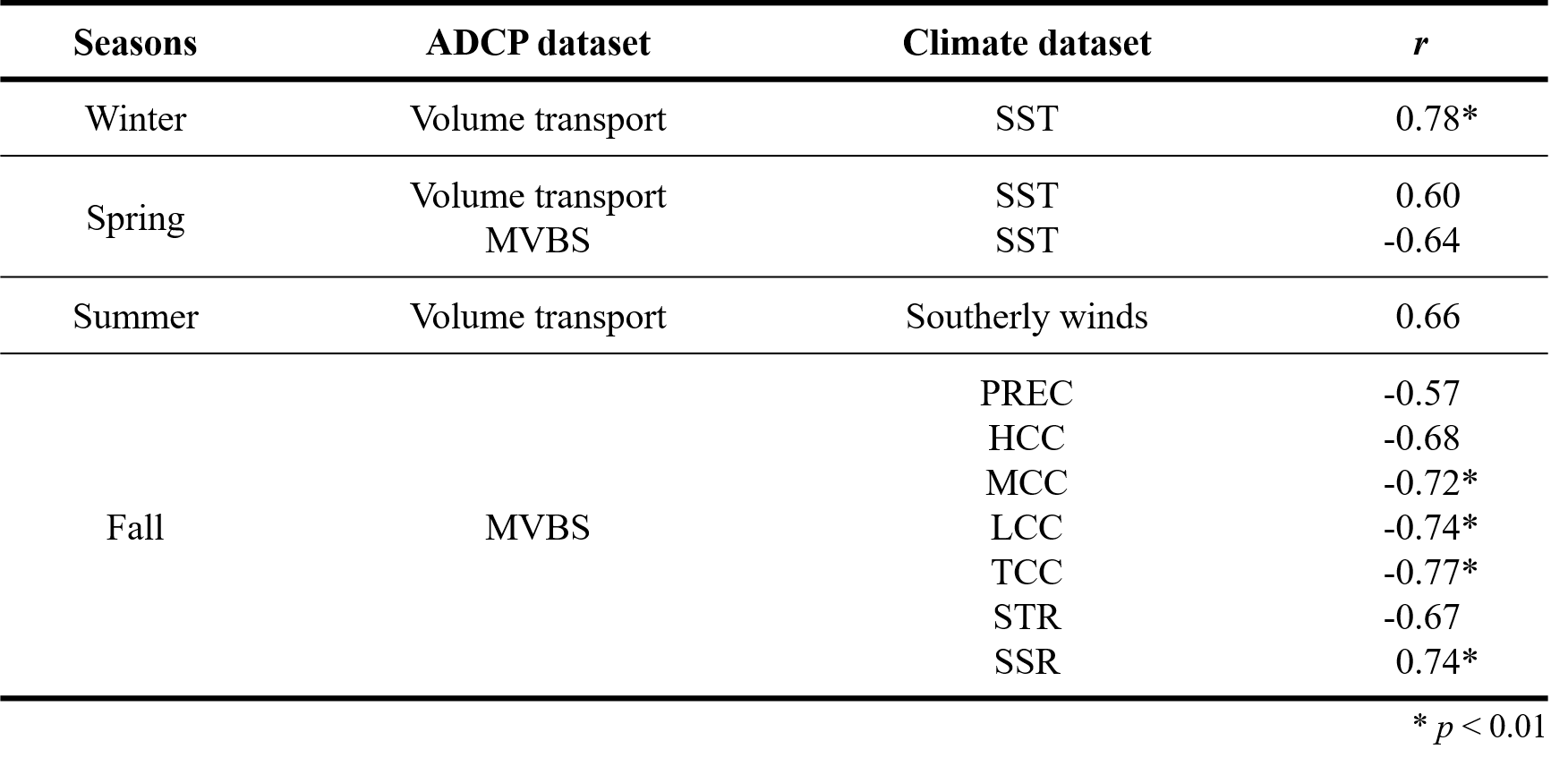


**Supplementary Table 3 | Correlations between NINO indices and other factors (volume transport, mean volume backscattering strength (MVBS) and climate data).** The correlations (*r*) were calculated for all datasets, but only significant (*p*<0.05) relationships are displayed. Bold letters are the significant relationships between volume transport (or MVBS) and climate data within same season (Supplementary Table 2), which are displayed in Supplementary Table 1. ADCP data includes volume transport and MVBS, and climate data includes sea surface temperature (SST), 10 m-above southerly winds, precipitation (PREC), high cloud cover (HCC), middle cloud cover (MCC), low cloud cover (LCC), total cloud cover (TCC), surface thermal radiation (STR), and surface solar radiation (SSR).


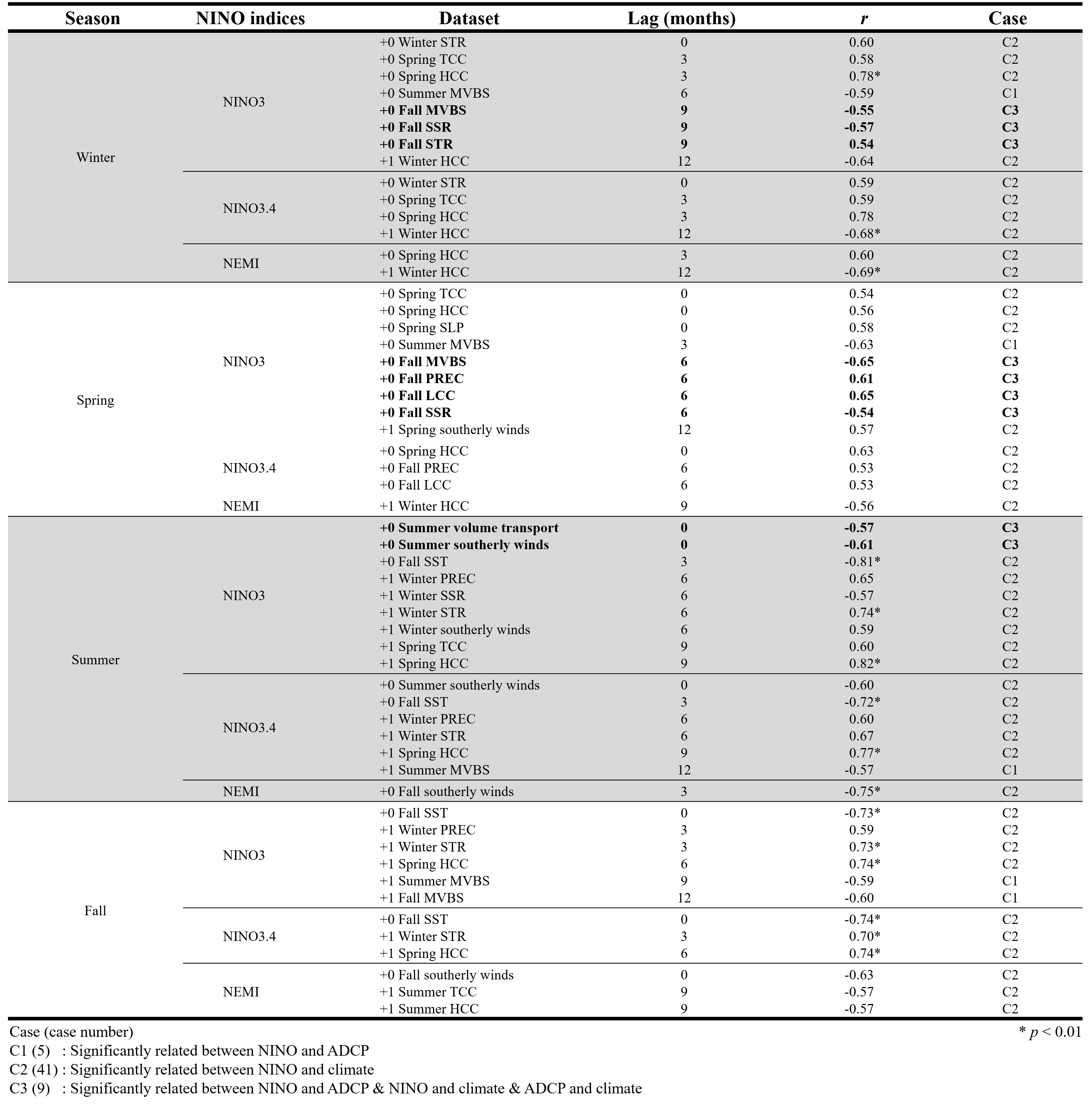


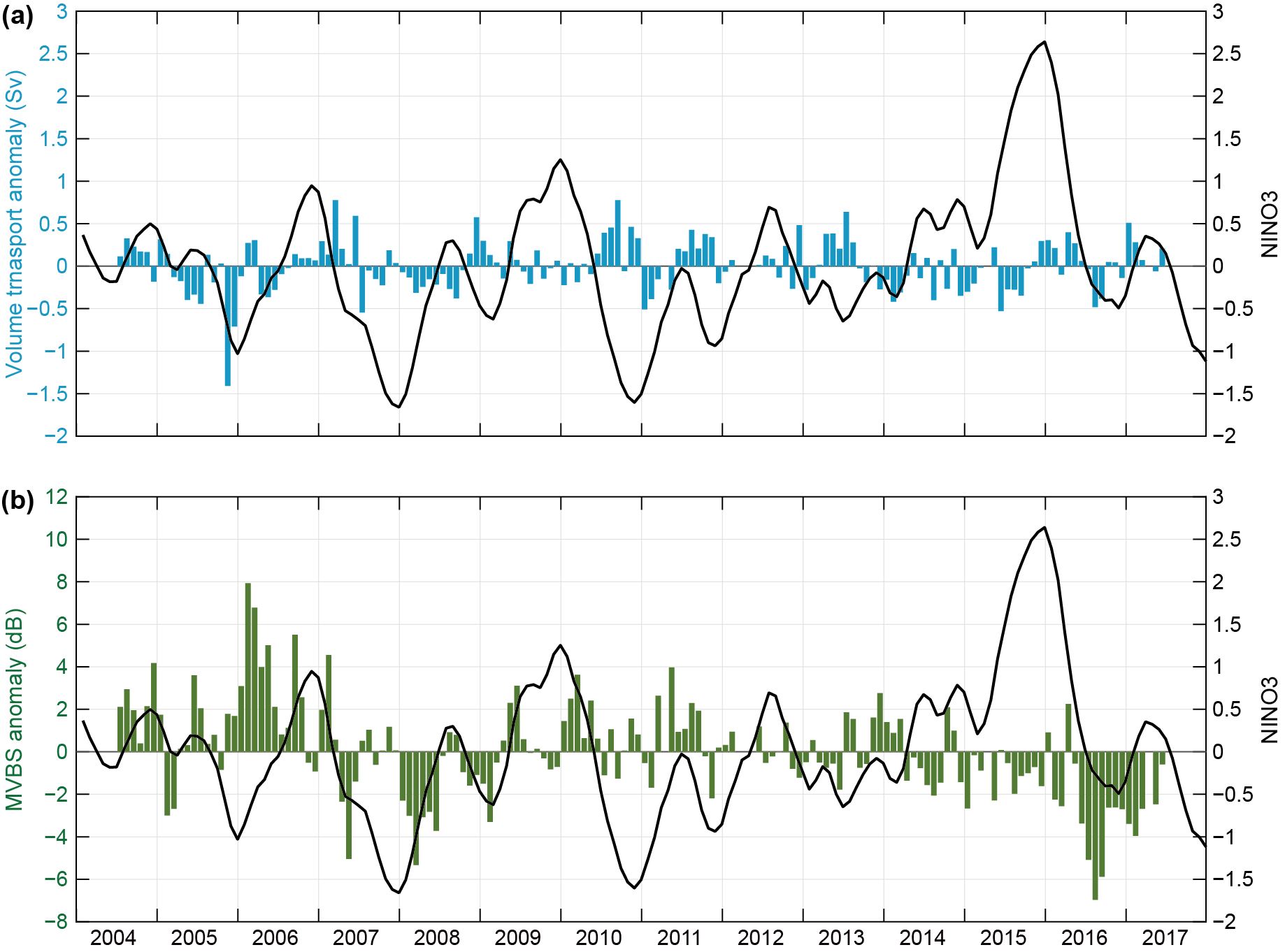


Supplementary Fig. 1 | Variability of oceanic and biological processes across the Korea/Tsushima Strait. a, Volume transport anomaly and b, MVBS anomaly derived from the *New Camellia* ADCP. NINO3 is the mean-removed and 3-month moving averaged SSTA over the 5°S–5°N and 150–90°W region ([https://www.esrl.noaa.gov/](https://www.esrl.noaa.gov/psd/gcos_wgsp/Timeseries/Nino3/)).


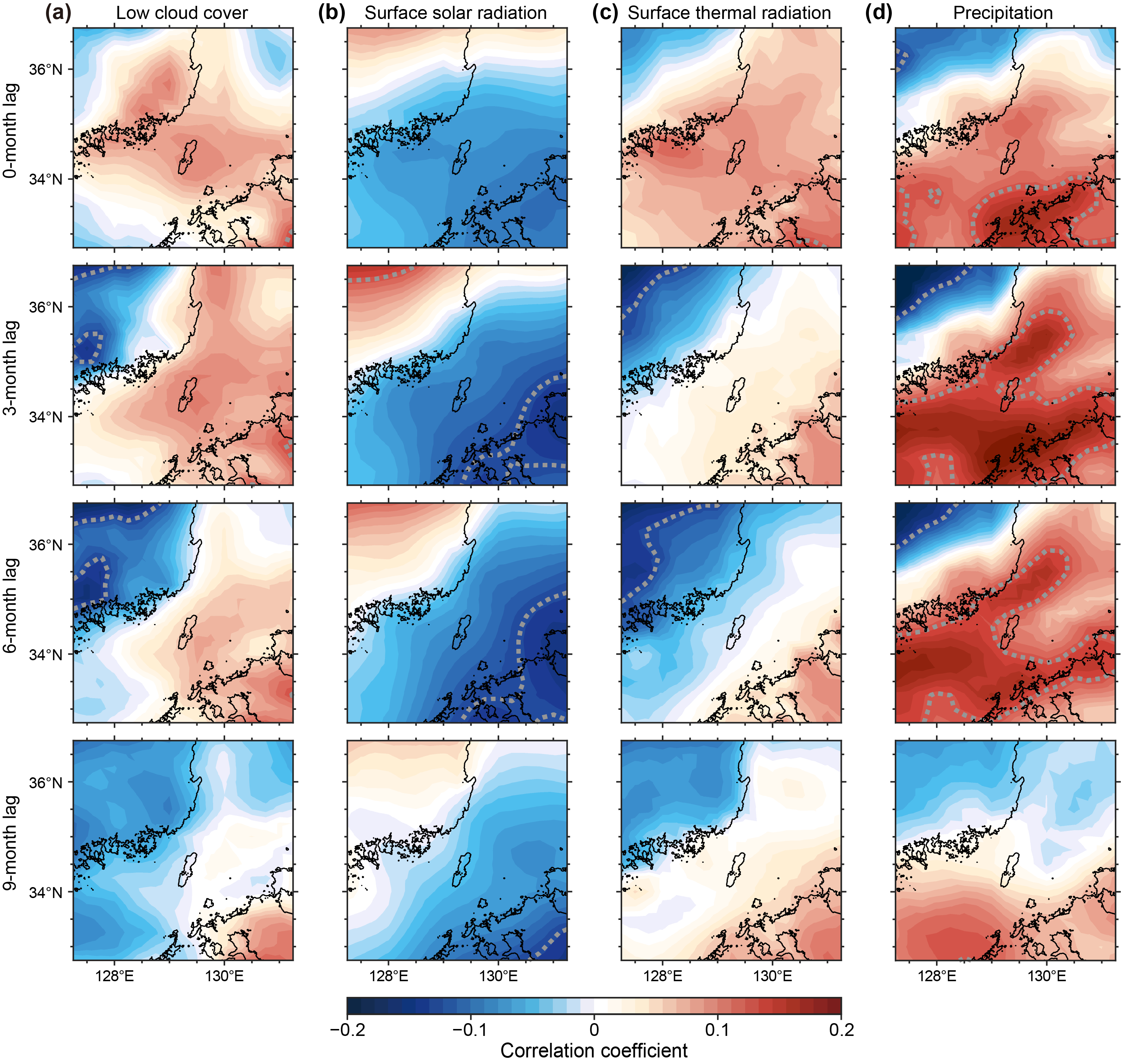


Supplementary Fig. 2 | Lag correlation between NINO3 and climatic factors. a, Low cloud cover, b, Surface solar radiation, c, Surface thermal radiation and d, Precipitation. The positive numbers indicate the months by which NINO3 leads the climatic factors. The figure was created using MATLAB (ver. 9.9.0.1592791 (R2020b) Update 5). The coastline is based on the SRTM30_PLUS dataset (<https://topex.ucsd.edu/>). Gray dashed lines denote statistical significance at the 90% confidence level between 110°E–130°W and 50°N–10°S.


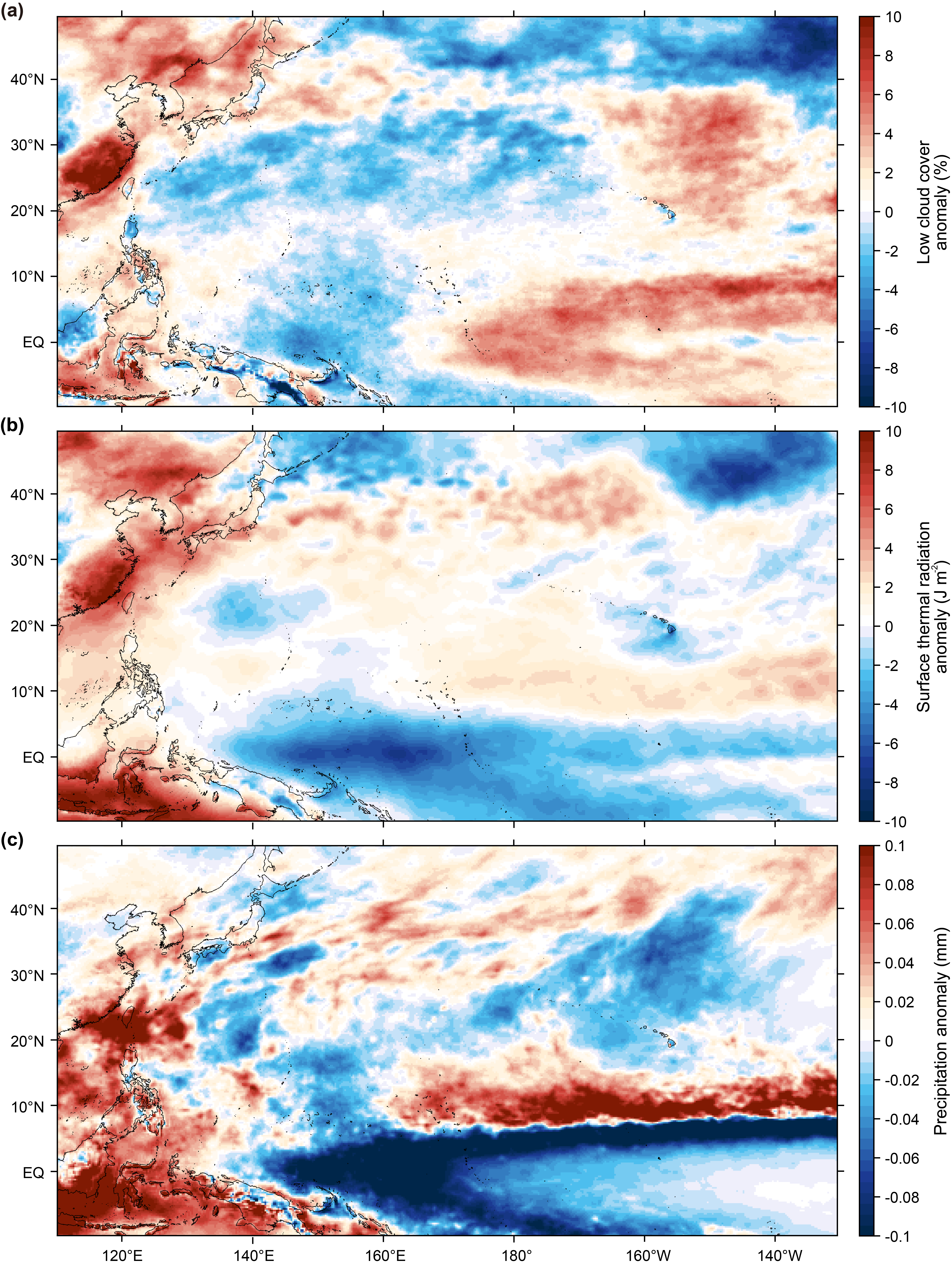


Supplementary Fig. 3 | Composite maps of the averaged anomalies of climate factors during the decaying fall of El Niño years (2009/10 and 2015/16). a, Low cloud cover, b, Surface thermal radiation and c, Precipitation. The figure was created using MATLAB (ver. 9.9.0.1592791 (R2020b) Update 5). The coastline is based on the ETOPO1 dataset (https://www.ngdc.noaa.gov/).


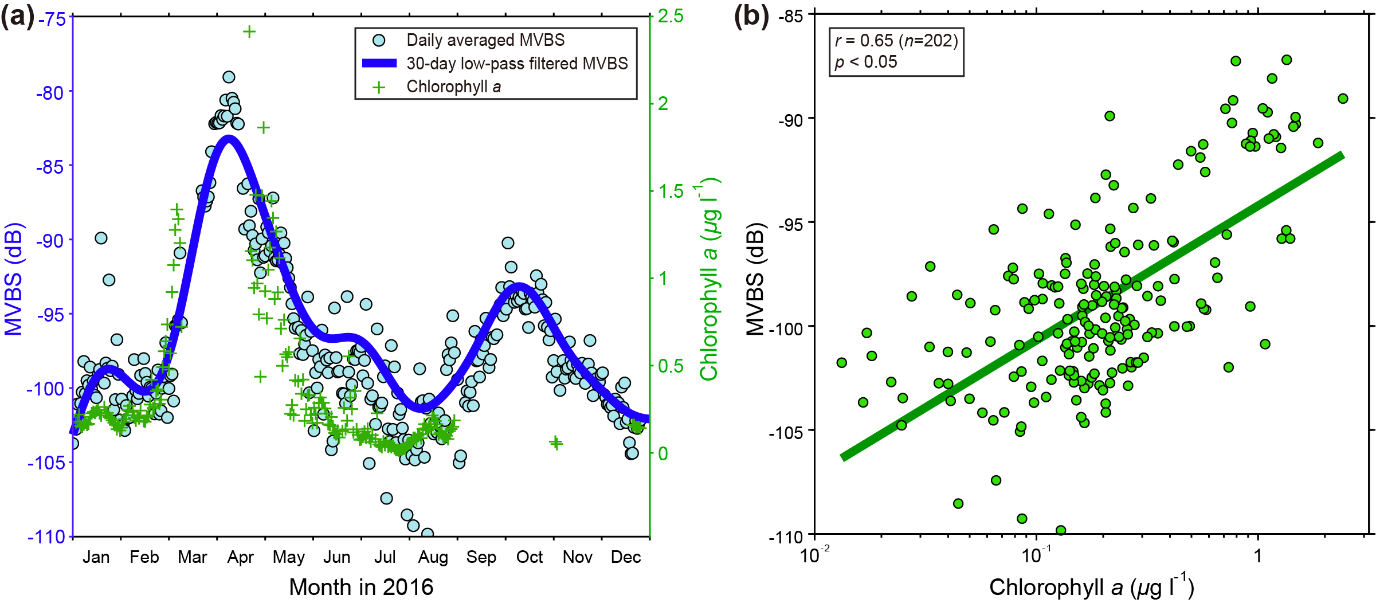


Supplementary Fig. 4 | Comparisons between the mean volume backscattering strength (MVBS) and chlorophyll-*a* concentration measured by *New Camellia* in 2016. a, MVBS and chlorophyll-*a* concentration show similar patterns with low values in winter and summer and high values in spring. b, Relationship between MVBS and chlorophyll-*a* concentration.


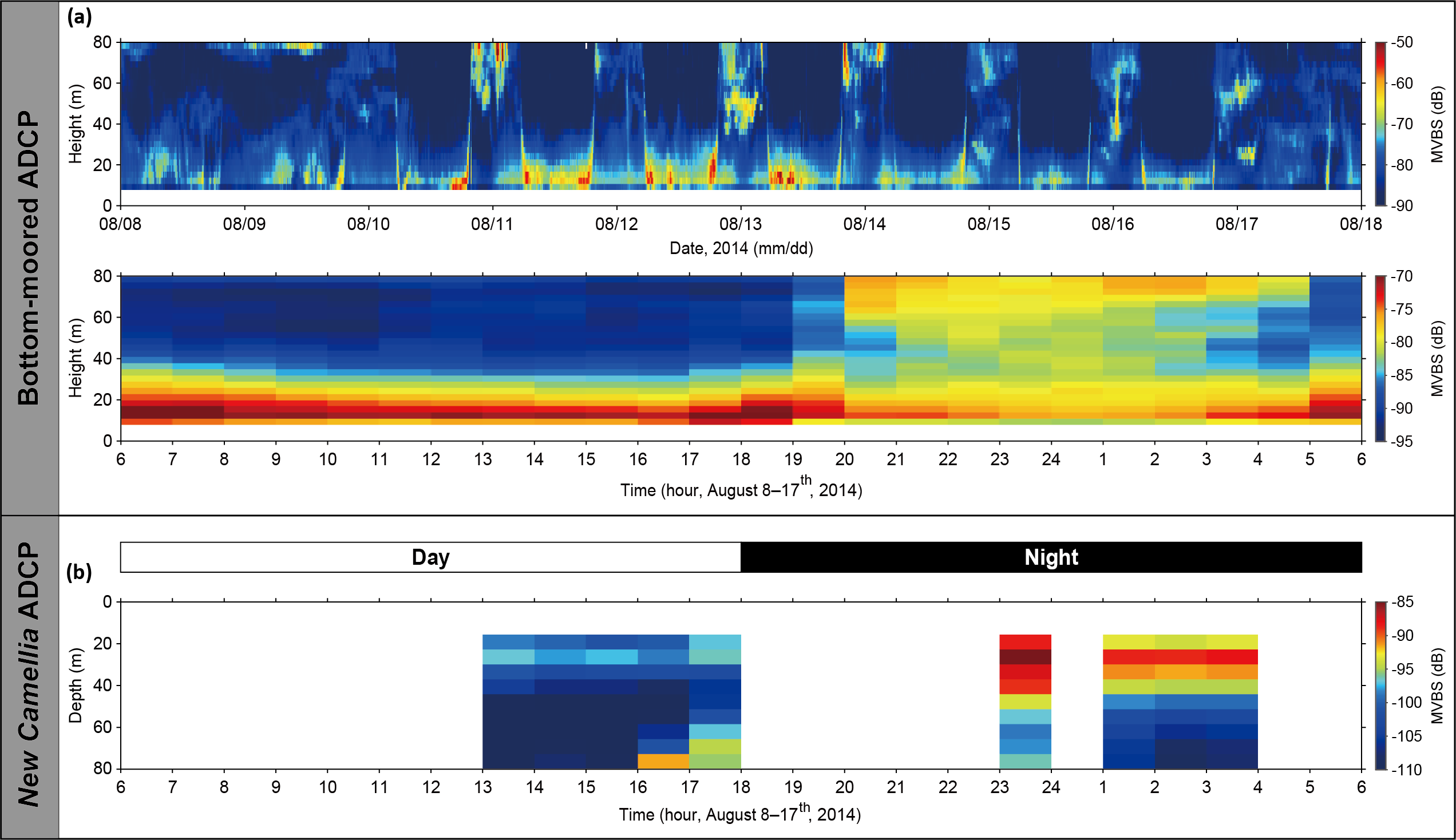


Supplementary Fig. 5 | Comparison between mean volume backscattering strength (MVBS) from bottom-moored and ferry-mounted (*New Camellia*) ADCPs. a, Time series and hourly-averaged MVBS from bottom-moored ADCP. b, Hourly-averaged MVBS from ferry-mounted ADCP across the western channel of the Korea/Tsushima Strait. A distinct pattern of diel vertical migration with high MVBS was observed near the bottom and the surface during days and nights, respectively. The missing data (white area in b) were due to the regular fixed operation schedule of the ferry.


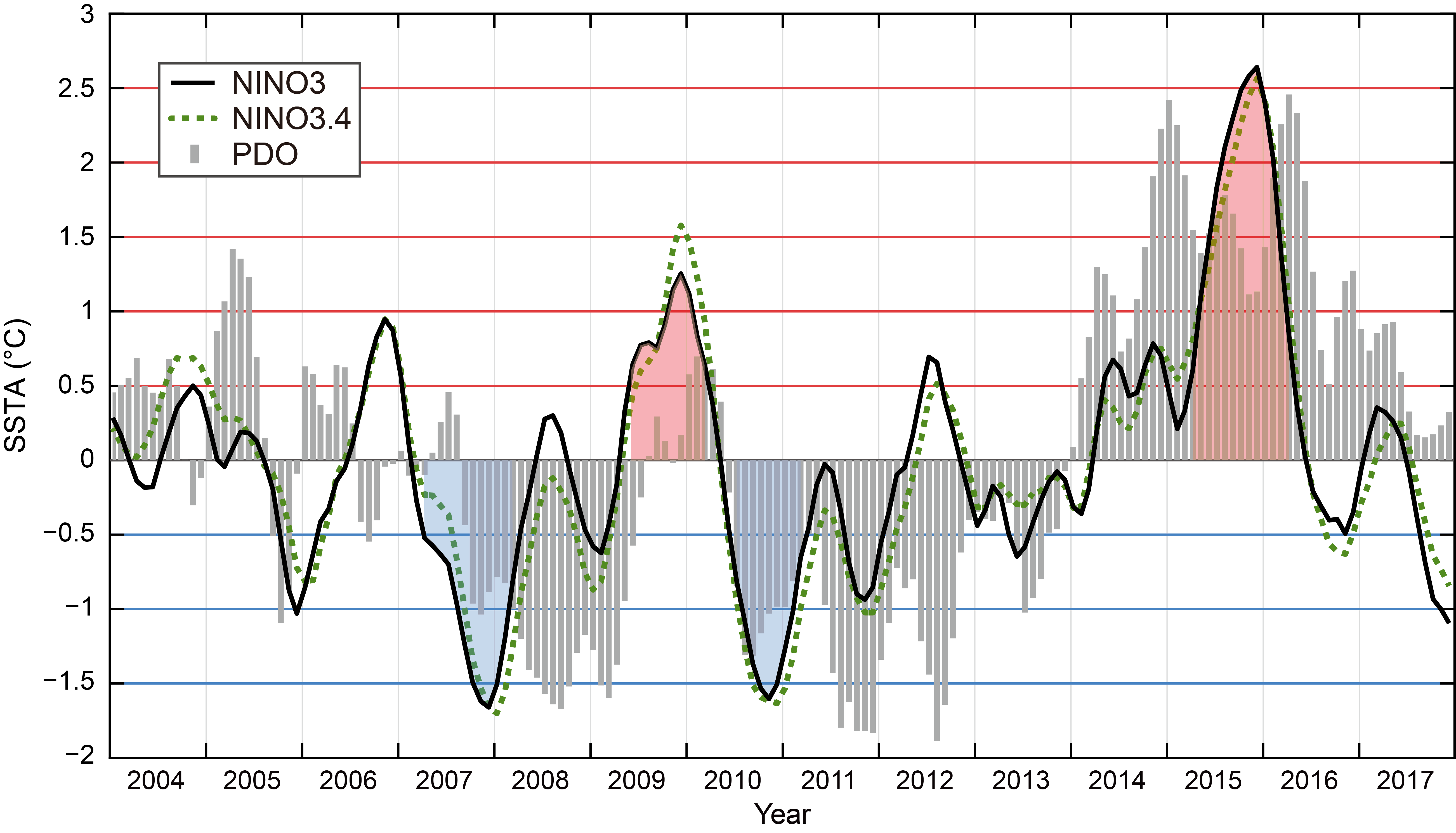


Supplementary Fig. 6 | Time series of NINO3, NINO3.4 and PDO. The El Niño (La Niña) events were identified by NINO3 exceeding (being less than) 0.5°C (-0.5°C) for more than 8 months^31^. The red and blue shaded areas indicate El Niño years (2009/10 and 2015/16) and La Niña years (2007/08 and 2010/11), respectively.
